# Supplementary material for: Translation initiation by the hepatitis C virus IRES requires eIF1A and ribosomal complex remodeling
Source: eLife. 2016 Dec 23;5:e21198. doi: 10.7554/eLife.21198 (PMC5238962; doi:10.7554/eLife.21198)
Supplement: Figure 4—source data 1. — Tables containing quantified band intensities from blots resulting from multiple biological replicates (n = 3). Blots were quantitated by densitometry, and the intensity of each eIF band was normalized to the rpS6 band. Graph shows the average of three independent experiments with the amount of each eIF bound to 40S subunit in the presence of WT HCV IRES set to 1. Errors represent one standard error from the mean. ND = not detectable. DOI: http://dx.doi.org/10.7554/eLife.21198.009 [file elife-21198-fig4-data1.docx]

**Unstressed (no treatment)**

|  | **No RNA** | **WT** | **IIIB** | **dII** | **GCC** | **CUG** |
| --- | --- | --- | --- | --- | --- | --- |
| **1A** | 0.7 ± 0.2 | 1.0 | 0.9 ± 0.1 | 0.7 ± 0.1 | 0.7 ± 0.1 | 1.0 ± 0.2 |
| **2α** | 1.6 ± 0.1 | 1.0 | 1.2 ± 0.1 | 1.3 ± 0.2 | 1.3 ± 0.2 | 1.5 ± 0.1 |
| **3B** | 1.1 ± 0.2 | 1.0 | 1.0 ± 0.3 | 1.3 ± 0.4 | 1.1 ± 0.2 | 1.0 ± 0.2 |
| **5B** | ND | 1.0 | 0.4 ± 0.1 | 0.5 ± 0.1 | 2.3 ± 0.1 | 1.5 ± 0.1 |

**Stressed with DTT**

| **eIF** | **No RNA** | **WT** | **IIIB** | **dII** | **GCC** | **CUG** |
| --- | --- | --- | --- | --- | --- | --- |
| **1A** | 0.9 ± 0.1 | 1.0 | 1.2 ± 0.4 | 0.3 ± 0.1 | 0.4 ± 0.1 | 0.7 ± 0.2 |
| **2α** | ND | ND | ND | ND | ND | ND |
| **3B** | 1.2 ± 0.0 | 1.0 | 0.7 ± 0.1 | 1.3 ± 0.3 | 0.9 ± 0.1 | 0.8 ± 0.1 |
| **5B** | 0.1 ± 0.1 | 1.0 | 0.2 ± 0.1 | 0.3 ± 0.0 | 0.5 ± 0.1 | 0.6 ± 0.1 |

**Treated with GMP-PNP**

| **eIF** | **No RNA** | **WT** | **IIIB** | **dII** | **GCC** | **CUG** |
| --- | --- | --- | --- | --- | --- | --- |
| **1A** | 1.2 ± 0.2 | 1.0 | 1.0 ± 0.3 | 0.7 ± 0.3 | 0.9 ± 0.3 | 1.4 ± 0.4 |
| **2α** | 3.5 ± 2.1 | 1.0 | 1.5 ± 0.4 | 3.9 ± 2.8 | 2.5 ± 1.3 | 4.1 ± 2.5 |
| **3B** | 3.1 ± 1.9 | 1.0 | 1.5 ± 0.3 | 4.0 ± 2.6 | 2.9 ± 1.4 | 3.3 ± 1.7 |
| **5B** | ND | ND | ND | ND | ND | ND |
